# The role of feed enzymes in maintaining poultry intestinal health

**DOI:** 10.1002/jsfa.11670

**Published:** 2021-12-07

**Authors:** Michael R Bedford, Juha H Apajalahti

**Affiliations:** ^1^ AB Vista Marlborough UK; ^2^ Alimetrics Group Ltd. Espoo Finland

**Keywords:** feed enzymes, poultry, digestion, microbiota

## Abstract

Gut health or intestinal health is frequently discussed without any clear definition as to its meaning. It is suggested that this should be defined as intestinal integrity and functionality as both are a pre‐requisite for the health of the intestine itself and the host. The health of the intestine is dependent upon a successful evolution of the absorptive capacity of the intestine, which in turn is influenced by the co‐evolution of the intestinal immune systems and the microbiota. Nutrient supply plays a significant role in this process and from the perspective of the microbiota this changes with age as the intestines and upper gastrointestinal tract (GIT) microbiota become more effective in nutrient removal. Feed enzymes play a significant role in this process. Phytases can improve digestion of minerals, amino acids and energy and as a result reduce the availability of nutrients in the lower intestines for the microbiota. Protease can have a similar effect with amino acid supply. Non‐starch polysaccharidases (NSPases) have a unique role in that they not only improve diet digestibility from the hosts perspective, thus limiting nutrient supply to the microbiota, but they also release soluble fragments of fibre from the insoluble matrix and/or depolymerize high molecular weight viscous fibre fractions in to smaller, more fermentable carbohydrate fractions. This results in a more favourable balance between fermentable carbohydrate to protein supply, a ratio which is deemed critical to maintaining good intestinal health. The dynamic nature of this complex evolution needs greater consideration if antibiotic free production is to succeed. © 2021 The Authors. *Journal of The Science of Food and Agriculture* published by John Wiley & Sons Ltd on behalf of Society of Chemical Industry.

## INTRODUCTION

Many papers discuss ‘gut health’ without actually providing a meaningful definition of what is meant by this statement. In some papers reference is made to the absence^1^ ^,^ ^2^ of pathogenic organisms or disease status or the presence of short‐chain fatty acids (SCFA; e.g. butyrate) that are presumed beneficial.^3^ In others, villus and crypt structure and size is referenced or the presence and integrity of tight junctions. Perhaps a more applicable definition should state that the intestines are fit for purpose, which means that the digestive and absorptive processes are unimpeded and the integrity of the intestine maintained, minimizing nutrient leakage or pathogen ingress. Indeed, intestinal integrity is clearly implied if digestion and absorption of dietary nutrients is to proceed efficiently, and thus perhaps the reference to ‘gut health’ should by default be more specifically referred to as intestinal integrity and functionality. Intestinal integrity and functionality are optimized when there is a balance between the host's needs (which includes its immune system), the diet and the intestinal microbiota. In essence, the balance is between the host and its environment, where environment refers to external (temperature, humidity, etc.) as well as internal (i.e. stage of intestinal development, microbiota, nutrient and antinutrient presence) environments. Dysfunction arises when this balance is lost. An increasingly important point to note is that there is more than just one equilibrium between host and the environment which can deliver intestinal integrity and functionality, and these equilibria change with age of the animal. For short‐lived animals such as the broiler chicken, gut health should not be viewed as a point‐in‐time measurement but a measure of how well the intestine has maintained its structure and function throughout the life of the animal. Performance is optimized when ‘gut health’ is maintained from hatch to slaughter which means the structure and function of the entire intestine, the microbiota inhabiting each section of the intestinal tract and the immune system are optimized during each stage of the growth cycle of the bird.

It is interesting to consider whether ideal ‘gut health’ is automatically linked to overall animal health and thus to good performance of production animals. It is obvious that poor ‘gut health’ will likely be associated with poor performance. However, it is possible that animals may have perfect ‘gut health’ but sub‐optimal performance. This could be the result of interventions designed to stimulate the immune system to counteract environmental challenges coupled with feeding lowered nutrient density diets to minimize excess nutrients for pathogens; factors that cannot be expected to optimize body weight gain and feed conversion efficiency. In well‐managed farms the need to prepare for and tolerate challenges may be minimal but when management and conditions are suboptimal more performance‐compromising safety measures are needed if gut health is to be maintained.

This paper reviews the factors which influence intestinal functionality and how such factors are driven by the diet, the environment in which the bird is raised and the age of the bird. The effects of exogenous feed enzymes on intestinal functionality will vary with age as a result of the rapid development and changes that occur post‐hatch and in the first few weeks of life. This article therefore considers the changes in the intestinal environments which the bird experiences and the effects such changes have on the impact of nutrition and feed enzymes on maintaining intestinal functionality and integrity.

## AGE – INFLUENCE ON INTESTINAL DEVELOPMENT AND NUTRIENT DELIVERY

The first few days after hatch sees a dramatic change in the nutrition of the chicken, with highly digestible, egg‐derived nutrient supply being replaced by dietary ingredients of a much more variable nutritive value.^4^ In response, there are significant changes in the gastrointestinal tract (GIT) as the dietary ingredients encountered demand the production of an array of digestive enzymes previously not required (e.g. amylases, alpha limit dextranases, maltase) coupled with a significant increase in the absorptive surface area of the small intestine.^5^ These changes are aimed at providing the greater capacity needed to deal with the transition from the assimilation of an easily digested and balanced nutrient supply to a far more difficult and nutrient‐imbalanced substrate. Moreover, this ‘new’ source of nutrients brings with it compounds which actively interfere with digestion and thus reduce the ability of the neonate to meet its demands for tissue growth. Additional stresses accrue as a result of the delay between hatch and placement which can significantly delay access to feed. Since the presence of feed in the intestinal tract is a significant stimulus for intestinal development and digestive enzyme output (both pancreatic and mucosal),^6^ such delays can impair the functionality of the intestine and thus its ability to extract nutrients from the diet.^7^ The ability of the bird to grow and develop is therefore reliant on rapid access to highly digestible feed such that the turmoil encountered in the switch from egg‐derived to diet‐derived nutrients is as seamless as possible. Regardless, it is clear that digestibility of nutrients in the newly hatched chick is, at best, poor but improves rapidly, commensurate and dependent upon the allometric development of the digestive tract and associated machinery.^8^

It is pertinent to note that the digestibility of protein is poorer than that of starch and fat at hatch and this ‘gap’ remains until approximately 10 days of age when amino acid digestibility appears to reach a maximum.^9^, ^10^ The greater demand for nutrients to sustain the far greater rate of growth of broilers compared with layers is not proportionate with the differences in their respective rates of intestinal and pancreatic development. Indeed, the concentration of digestive enzymes in the pancreas and small intestine are not different or perhaps even err in favour of the layer.^11^ Consequently, it has often been stated that the rate of growth of the neonatal bird, in particular the rapidly growing broiler, may well be limited by intestinal development and digestive enzyme output, even under ideal conditions. Even marginal stresses can further exacerbate these problems. Handling, for example, can reduce amylase output from the pancreas, a problem which presents an opportunity to supplement endogenous enzyme deficiencies with exogenous enzyme application.^12^, ^13^ As a result, the neonatal chick is often thought to be the most responsive to exogenous enzyme application as a result of its knife‐edge digestive capability. Furthermore, this renders it more susceptible to the negative effects of dietary anti‐nutrients which hamper the digestion process. As the bird ages the intestine becomes more competent and as a result deficiencies in enzyme output and intestinal absorptive capacity are thought to wane, making the environment of the small and large intestine more stable and conducive to establishment of an equilibrium between the digestive capacity of the host and its own nutritional requirements.^9^, ^10^ This is coupled with an equilibrium between the host and the microbiota,, effectively establishing a physiological homeostasis which optimizes intestinal health and thus growth rate and efficiency of the bird. Exogenous enzyme addition could therefore be considered to become less important with bird age but evidence suggests there are considerable gains to be made through continued use of certain enzymes, some of which clearly influence the development of the microbiota in later life. Thus, it is not simply the effect of exogenous enzymes on digestibility of nutrients which contributes to intestinal health (which is undoubtedly the case as a good supply of nutrients is required to fuel intestinal growth) but also the effect they have on delivery of nutrients to the microbiota in both the small and large intestine. This latter effect may be of greater consequence than the former, particularly in adolescent birds where the microbiota is still in flux. The parameters which demonstrate optimal gut health are clearly linked with the challenges the intestine has to deal with which change with age. As such it is probably not possible to set a single standard or criteria of gut health which remain relevant throughout the life of the bird.

## FACTORS INFLUENCING THE ESTABLISHMENT, DEVELOPMENT AND IDENTITY OF THE MICROBIOTA

Just before hatch the intestine of a chick has a very minimal microbiota. In nature, the newly hatched birds get their first bacterial inoculum from the ‘mother hen’, usually the caecal droppings surrounding the nest. It is important to note that such an inoculum is composed of microbes pre‐adapted to intestinal conditions and the diet at hand. In fact, coevolution of the bird and its intestinal microbiota may have been going on for millennia to reach the ideal symbiosis between the two. In modern hatcheries, the hatching eggs are isolated from the mother hens and even surface‐sterilized. In practice this means that any coevolution of the bird with its microbiota loses its meaning and the finely tuned symbiosis fails to materialize. Instead, the initial bacterial inoculum ingested by the newly hatched chick may be totally foreign to the intestinal tract. Still, it is the initial inoculation that determines which subsequent species can establish in the individual bird. This is governed by what species dominate at the hatchery, during handling and transit to the shed for rearing, in the shed itself, the water and feed offered, and the status of the immune system which responds to a multitude of environmental and nutrient stressor inputs.^4^ The nutrients which fuel the growth in population and diversity of the microbiota in the distal intestine are comprised of those that the host has failed to digest and absorb itself. Thus, perturbation of digestion of the host has consequences for the development of the microbiota. This in turn influences intestinal integrity and function as a consequence of the environment that is created by both the products and presence of specific members of the microbiota. The structure of the microbiota is therefore very much dependent not only upon the nutrient resource available to it but also the identity of the species that have managed to establish from the surrounding environment. The number of factors influencing this ‘start point’ is huge and their interaction means that there are almost an infinite number of possible microbiotas when viewed on the lowest level of taxonomic hierarchy [operational taxonomic unit (OTU)] basis, thus a view of functionality rather than individual inhabitants of the intestinal tract is seen as being more informative.^14^ Regardless, the combination of all of these factors determine the ‘intestinal environment’, a term that will be used throughout this text.

While aerobic microbes produce mainly carbon dioxide as the end product of their metabolism, the main metabolic end‐products of intestinal anaerobic bacteria are organic compounds, the variety of which is determined by the substrates available and the types of microbes present. In the GIT the products are typically SCFA, biogenic amines, ammonia, indoles, etc. that are produced in quantities dependent upon the composition of the diet and more importantly the fraction remaining undigested/unabsorbed by the host. All of these bacterial products have been shown to have some influence on the structural integrity and function of the intestine.^15^, ^16^, ^17^, ^18^, ^19^, ^20^ Clearly the goal for optimum intestinal health is to optimize the beneficial microbial metabolites and minimize or eradicate the irritants and toxins.^21^ At present we can identify the beneficial (e.g. SCFA, especially butyrate) and likely detrimental metabolites (e.g. amines, ammonia, indoles) but the desired minimum or maximum concentrations of either are not known. In this regard, the most desirable outcome may well be for minimum protein and optimal carbohydrate fermentation, with one of the most desirable end products being butyrate as a result of its multiple beneficial effects on enteric and systemic health and metabolism.^21^, ^22^, ^23^, ^24^ This seems to suggest that the goal is to aim for a specific microbiota in every single bird, in every flock and thus optimize performance. The problem with this concept is that the microbiota is not a constant and its structure, as intimated earlier, depends on a multitude of factors. Consequently, the structure of the microbiota when viewed at the OTU level is not particularly meaningful as it is the result of the combination of specific conditions encountered by the individual bird. Indeed, even when the same feed, same breeder flock as a source of hatching eggs, same barn and pens were used in three successive trials, the resultant caecal microbiotas were totally different and independent from one another^25^ despite the standardization of many of the factors thought to influence microbiota structure. As a result, there is no such thing as a fixed or target microbiota.^14^ The optimum will vary depending upon the conditions under which the bird is grown. In fact, there are perhaps many different structures of a microbiota at any given age which can perform the same functions as far as the host is concerned. The reason is that there are multiple species of bacteria that can ferment a particular substrate and produce the same metabolites and as a result yield the same host response.^14^, ^21^ Thus, it is not simply the identity of the organisms present in the microbiota which is essential for intestinal integrity and function, but also their metabolic functions and the metabolites that they supply.

## CHANGES IN THE MICROBIOTA AS GOVERNED BY CHANGES IN NUTRIENT SUPPLY

In newly hatched birds the identity of the primary colonizers of the intestine is environment dependent and/or random, unless chicks are intentionally inoculated with a starter culture. When the use of culture independent methods came into use it was possible to follow the rate of colonization and the time it took for the small intestine and caecum to reach the maximum bacterial density. When studying bacterial densities post hatch by flow cytometry it was found that as early as 1 day after hatching, the total bacterial density in the ileum had reached 1E8/g and in caecum, 1E10/g.^26^ Two days later the densities had exceeded 1E9/g in the ileum and 1E11/g in the caecum and the numbers do not increase further over the next 30 days.

Although the total density of bacteria changes little beyond 2 days of age, significant shifts take place in population structure. The microbial community seeks an equilibrium where each member exists for a reason, being the fittest and/or having a characteristic which is fundamental for the survival of the community. In many studies, shifts in microbiota composition are detected but the fundamental reasons for the shifts are unknown. As an example, the multiple species of *Lactobacillus* are often considered as one group, beneficial for intestinal health. In a study, four commercial broiler farms were sampled when birds were 1 or 5 weeks of age.^27^ Resident lactobacilli in the crop were studied and the most common species were *L. salivarius*, *L. crispatus* and *L. reuteri*. In young birds, *L. reuteri* dominated regardless of the farm or the brand of commercial feed used. However, as the birds aged, the abundance of *L. reuteri* dropped in all farms.^27^ Although the reason for such a shift is unknown, it may have an impact on intestinal health since *L. reuteri* is the only heterofermentative *Lactobacillus* species, meaning that it produces different metabolites than the other, homofermentative, lactobacilli. Thus, the proportionate production of lactate will increase relative to other SCFAs as *L. reuteri* is replaced by other species with likely consequences on the environment of the crop.

In the very young animal, a proportion of almost all nutrients will bypass the small intestine and present themselves in the large intestine where they can either be voided as faeces or fermented by the large intestinal microbiota, which from a functional viewpoint is dominated by the caeca. As the bird ages and bacteria populate the upper intestine, those nutrients which are more readily digested by the host and/or fermented by the small‐intestinal microbiota such as monosaccharides, free amino acids and readily digested peptides followed by highly digestible proteins and starch are progressively stripped out from the large intestinal supply with the result that the large intestinal microbiota is exposed to a more resistant range of nutrients.^9^, ^10^ As the microbiota in the crop and small intestine develop they begin to remove some of the fermentable oligosaccharides and more soluble polysaccharides leaving the large intestine with progressively ‘hard to digest’ fibre such as linear β‐(1‐4)‐glucans and arabinoxylan. In the small intestine, the diversity of microbiota is relatively low, which is typical of an environment rich in nutrients and with a high rate of metabolite production. In environments such as the caecum where easy substrates (e.g. simple sugars and amino acids) are depleted, a more complex microbial community settle. Microbes tackle difficult substrates as a community and it is more difficult for a single bacterium to take over. Thus, the large intestinal microbiota has to adapt and diversify as its food supply becomes constricted and in a manner that does not facilitate disruption in gut integrity through production of toxic metabolites. It is clear from analysis of the caecal microbiota over time that there is a transition from species able to ferment starch, protein and many other sources of carbohydrate towards one which is almost exclusively targeting fibre fermentation.^25^, ^28^, ^29^, ^30^ Readily fermentable carbohydrates such as starch and saccharides most often result in production of lactate in both the ileum and caecum,^31^ whereas fibre tends to promote more of an acetate and butyrate fermentation in chickens. If this transition to fibre fermentation is too slow then it may increase the likelihood of an imbalance between fermentation of carbohydrate relative to protein and as a result a slide towards problems related to production of toxic products of putrefaction. Evidence to support this transition has recently been reported in a study investigating daily changes in the caecal microbiota from 3 to 35 days of age and suggests that there is indeed a rapid evolution and increase in diversity in the caecal microbiota up to 12 days of age, this being driven by available space and food.^30^ After 20 days of age the authors noted a stabilization in the microbiota in both diversity and abundance of species which was driven by the environmental conditions presented by each host chicken. In other words, the substrate and space available in the caeca were more or less constant after 20 days of age and hence the microbiota stabilizes into a structure best suited to the conditions which exist.

It was noted that opportunistic *Campylobacter* spp. appeared in 25% of the birds by day 16,^30^ possibly as a result of the ‘turmoil’ created by the loss of or imbalance between substrates entering the caeca and as a result a disruption in the environment (concentrations of SCFA, metabolites, toxins, redox potential, for example) which reduces resilience against opportunistic invaders. Similar work which evaluated broiler faecal microbial DNA (as a proxy for ileal populations) showed a similar rapid increase in diversity to 10 days of age followed by a period of significant adaptation between 14 and 21 days of age where perturbations of the ileal microbiota were most likely,^32^ and thus interventions most likely to succeed. The change in nutrient supply and environmental conditions during this period likely contribute to the explanation of these observations. Thus, changes in dietary ingredients and nutrient digestibility with age need to be considered when reviewing microbiota shifts.

One further consideration with age is that as a bird grows it is common practice to switch from higher protein, lower energy diets to lower protein, higher energy diets. The transition from one diet to the other not only changes nutrient density but as a consequence ingredient composition changes to affect these nutrient targets. Cereal inclusion rates typically increase and protein meals (e.g. soybean meal, rapeseed meal) typically decrease in content with each progressive diet change. Furthermore, it is well known that digestibility of ingredients varies from batch to batch.^33^, ^34^, ^35^ Even if the ingredient composition of the diet did not change the fact that a diet made several weeks later would contain different batches of wheat or corn, soybean meal and other macro ingredients, for example, would mean that the digestibility of energy, protein and the fibre composition of the two temporally‐separated diets would be different. Thus, the combination of changes in ingredient batch and inclusion rate between different diet phases would be expected to create an initial ‘shock’ on the ileal and caecal microbiota as it struggles to adapt to a sudden change in nutrient availability. The concept that such a problem might transpire is supported by recent data which shows major changes in abundance (> 2 log fold change) in a high proportion of species present in the caeca directly following the change from starter to grower, and from grower to finisher phase diets.^30^ Clearly such changes represent an opportunity for pathogens to gain a foothold whilst the microbiota is not stable, particularly if the flow of protein to the large intestine is increased. In consideration of the discussion earlier, presentation of a stable supply of fermentable substrate during diet change may become a strategy to mitigate such changes in the future. Indeed, maintenance of intestinal health throughout the entire life of the animal is dependent upon a gradual rather than abrupt change in flow of fermentable substrates into the ileum and caeca.

## THE IMMUNE SYSTEM – ITS INTERACTION AND RELEVANCE WITH INTESTINAL HEALTH

Nutrient flow thus appears to be of significance in determining how the health of the intestine and the structure of the microbiota will develop. Optimizing gut health and feed conversion efficiency requires a ‘beneficial’ microbiota (which will vary between individuals and with age) and as a result a minimally activated immune system such that inflammatory responses and all the associated disruption of intestinal integrity and function and hence growth rate and efficiency is kept to a minimum. Pathogen recognition by the immune system is known to play a significant role in depressing performance though activation of energy‐costly inflammatory responses whether the challenge is acute or chronic. Such a response can result in loss of intestinal integrity and an increase in permeability of the intestines. This can have significant consequences as nutrients fail to be absorbed efficiently, some are lost from sloughed cells and some leaked through failed tight junctions.^21^ This provides further habitat and nutrients for opportunistic pathogens and is thought to be the process by which *Clostridium perfringens* establishes in the small intestine.^36^ Further losses occur as a result of divergence of resources away from growth towards the intestinal inflammatory response and the systemic inflammation as a result of leakage of toxins and metabolites towards the liver, which can precipitate metabolic disorders.^23^, ^37^ The immune system will also respond to excesses of certain nutrients or metabolites which can invoke inflammatory responses with similar repercussions.^23^ The goal is to minimize inflammatory responses such that intestinal health and thus growth rate and efficiency are optimized, whilst maintaining the immune system on sufficient alert to deal with significant disease challenges should they occur. The principles which govern whether a feed enzyme can play a role in whether the microbiota and/or nutrient flow will be beneficial or not are dealt with in the following section. Exogenous enzymes not only can restrict the flow of protein, starch, lipids and minerals simply through enabling a more efficient digestion by the host but they can also deliver nutrients as a result of hydrolysis of indigestible fibre to more fermentable oligosaccharides for example.

The use of prophylactic antibiotics was effective in reducing and in many cases eliminating the consequences of imbalanced or excessive flow of nutrients into the ileum and particularly the caeca by directly limiting the ability of pathogens to take advantage. Removal of these products significantly increased the opportunities for microbial overgrowth and with it the pressure on both the immune system and intestinal health increased, bringing to the forth the need to manage nutrient flow with far greater vigour.

## NUTRIENT FLOW ‘MANAGEMENT’

Enzymes have been used in feeds for production animals for decades, but the range of enzymes and the rationale for their use has broadened over the years. The main purposes of enzyme use are the elimination of anti‐nutrients of feed origin and improvement of the efficiency of nutrient capture from feed. The most commonly enzyme‐targeted anti‐nutrients and substrates in poultry rations are;

1. Non‐starch polysaccharides (NSP)
2. Phytic acid
3. Starch
4. Protein

The relevance of each of the above changes with the aging of both the intestinal tract and its resident microbiota.

### NSP enzymes

#### Non‐starch polysaccharidases (NSPases) and their role in reducing nutrient flow to the microbiota

NSP can present two main problems and one opportunity. The first problem is that of increased intestinal viscosity, which delays the rate of digestion and the second is nutrient encapsulation, which effectively reduces nutrient availability from the grain. A thorough review of these concepts and in particular a critique of the relevance of the nutrient encapsulation effect has been published recently.^38^ Regardless of the relative importance of each of these mechanisms, the consequence of each is that undigested nutrients that escape the upper digestive tract where bacterial numbers are limited effectively become the culture medium in the lower intestine, where bacterial populations are much denser. Protein and starch digestion are compromised as expected by both viscosity and the nutrient encapsulation effect and as discussed later such issues have consequences for both the host and the microbiota. The digestion of fat, however, is compromised most as a result of feeding high viscosity diets, especially if the fat is saturated and requires emulsification.^39^ This problem is exacerbated as a consequence of the negative effect on the microbiota of feeding a diet which is both viscous and high in saturated fat content.^39^ When viscosity was reduced by application of a xylanase there was a significant reduction in mucosa‐associated enterococci and other Gram‐positive cocci in the duodenum, jejunum and ileum. Moreover, this effect was greatest in a tallow *versus* a soy oil supplemented diet.^40^ In addition, excess undigested fat in its own right is thought to directly invoke inflammatory responses in humans and increase permeability of the small intestine as a result.^23^ Since viscous diets markedly reduce the digestibility of saturated fats to a far greater extent compared with unsaturated fats, it is possible that intestinal inflammation is greater with saturated fats and thus the environment for colonization by pathogenic bacteria enhanced.

Viscosity^41^, ^42^ and the benefits of viscous NSP hydrolysis seem to be greatest in the young chick^43^ and thus the reduction in nutrient bypass (i.e. the increment in nutrient removal) on enzyme supplementation would be expected to be greatest in younger birds. Since viscous diets also make the small intestine more anaerobic, responses to enzymes addressing this effect would once again be expected to be greater in younger birds. The combination of these effects subsequently influence which species can or cannot thrive by defining the intestinal environment. As a consequence of increased anaerobiosis and reduced redox potential, strictly anaerobic bacteria which typically inhabit the caecum and colon and are normally excluded from the small intestine due to its oxygen and redox potentials, can establish and become part of small intestinal microbiota.

Age‐related changes in the abundance of various microbes reflect nutrient and space availability as previously mentioned,^30^ the former being very much influenced by intestinal viscosity and the presence of endosperm cells which are intact and therefore opaque to digestive enzymes. As a result, the application of enzymes which address the problems of viscosity and nutrient encapsulation result in a significant advantage for the host, in that starch, protein and fat digestibility is enhanced and as a result more nutrients are available for maintenance and growth and a potential inflammatory agent removed. For the microbiota, the consequences are magnified in that the restriction on flow of nutrients to the ileum and caeca can be much greater than the increment in nutrient extraction by the host. As noted previously,^44^ increasing ileal digestibility of protein from 67 to 73% by inclusion of a xylanase in a wheat‐based diet results in almost 10% more protein being absorbed by the host, but it also resulted in a 20% reduction in protein flow out of the ileum. If viscosity is overwhelming then digestion of starch and protein may be markedly delayed, and coupled with an anaerobic environment this can result in as much as a ten‐fold increase in ileal fermentation.^45^ This was entirely overcome if the viscous agent (in this case a purified soluble arabinoxylan) was enzymatically degraded. Thus, NSP destruction can have significant benefits on both the ileum and caecum through accelerating the ability of the bird to remove readily digestible nutrients from the diet, restricting their flow to both sections of the intestinal tract. This forces the microbiota to change and adapt to a more ‘fibre‐centric’ fermentation, away from readily fermented starch and protein, as fibre becomes the only significant carbohydrate source at their disposal. Provided protein flow is reduced at a greater rate than that of starch, and the ability of the microbiota to ferment fibre picks up rapidly enough, then the balance between SCFA and putrefactive compound production is tipped in favour of the former and intestinal health is improved. The time required to develop a competent, fibre‐degrading microbiota is thus critical in this process and is discussed in the next section.

The caecum is inhabited by relatively few large bacterial families; among those perhaps the most abundant are *Ruminococcaceae* and *Lachnospiraceae*, earlier referred to as Clostridium clusters IV and XIVa. These families, characterized by their capacity to degrade plant polysaccharides and produce butyrate, belong to the bacterial order *Clostridiales*. Despite the ominous name of the order, most bacteria assigned here are non‐pathogenic or considered highly beneficial. *Ruminococcaceae* and *Lachnospiraceae* families are diverse and still largely uncharacterized (for review see Rychlik^46^). Consequently, the identity of the species within these families and the metabolites produced are often unknown as is their impact on intestinal health, and moreover this will vary from trial to trial.^14^, ^25^, ^30^, ^32^

#### Non‐starch polysaccharidases (NSPases) role in nutrient provision to the large intestinal and caecal microbiota

In addition to their ability to limit the flow of readily fermentable nutrients such as starch and protein to the microbiota as discussed earlier, non‐starch polysaccharidases (NSPases) are also capable of providing fermentable oligosaccharides as a result of their activity on polymeric NSP. This ‘prebiotic mechanism’ has been proposed more than 20 years ago^47^ but has never been considered as important as the viscosity or nutrient encapsulation mechanism until recently.^38^ As an endo‐acting NSPase randomly cleaves the backbone of its target NSP, it will generate large polymeric fragments which will become progressively smaller with successive hydrolytic events. The length of the oligosaccharide generated (e.g. an arabino‐xylo‐oligosaccharide) determines which species can utilize it as a substrate and what end products are produced.^48^, ^49^ Some work even suggests that the oligosaccharides generated by endo xylanases can directly influence the immune system by binding to toll‐like receptors.^50^ If the relevant de‐branching and exo‐activities are present at relevant dosages then the oligosaccharides could ultimately be reduced to its constituent sugars, effectively removing the beneficial prebiotic effects. Whether this occurs in the confines of the intestinal tract is unknown but it may play a role in the variability in responses noted in the literature. Nevertheless, there is evidence to suggest that in the small intestine and possibly the caecum the presence of an NSPase results in increased lumenal quantities of oligosaccharides, many of which are prebiotics.^51^, ^52^ Several pieces of research have shown that feeding these oligosaccharides directly results in changes in the ileal and caecal microbiota^53^, ^54^ which are somewhat similar to that noted when an NSPase is used. It therefore seems that oligosaccharide generation by the use of an NSPase is at least part of the mechanism of action of this class of enzymes. Such an activity would be especially welcome during the critical phase between 12 and 20 days of age when the fermentable substrate limitations in the ileum and more so the caeca become critical and the resultant reduction in the flux of substrates into the caeca rapidly changes microbiota structure and numbers. If the fibre degrading microbiota were established before nutrient restriction commences then the changeover from undigested starch and protein to fibre digestion would presumably be less erratic. As part of the prebiotic mechanism it has been suggested that supplying the lower intestine with arabinoxylan‐oligosaccharides (AXOS) results in the generation of significant quantities of SCFA which can be used as an energy source by the host. Generally, the species present in the ileum are various lactic acid bacteria (LAB; representatives include *Lactobacillus* spp and *Enterococcus* spp.), some of which can utilize simple oligosaccharides as substrates and produce lactate and acetic acid as the end products, whereas those in the caeca belong to *Ruminococcaceae* and *Lachnospiraceae* families and tend to produce acetate and butyrate as the main SCFA. Indeed, microbial utilization of xylo‐oligosaccharides (XOS) *in vitro* initially focussed on screening LAB and *Bifidobacteria*, and it was noted that different strains varied significantly in their ability to use XOS of varying chain‐length and degree of substitution. *Bifidobacteria* were found to prefer short un‐substituted XOS, with double substituted AXOS from wheat being particularly poorly utilized.^55^ The ideal situation is to stimulate both the ileal and caecal microbiotas directly and selectively to optimize stability, with the added benefit that the lactate generated in the ileum, if not absorbed and used as an energy source, will enter the caeca where lactate utilizers such as representatives of the genera *Eubacterium*, *Anaerostipes*, *Veillonella*, and *Megasphaera* will further ferment it to butyrate and/or other SCFA.^56^ Indeed such ‘cross‐feeding’ is of particular value to the host since the removal of lactate prevents damage to the intestinal mucosa and the final product, butyrate, is very much involved in maintaining intestinal health as noted earlier. It is interesting to note also that the ileal microbiota may be more adept at using shorter oligosaccharides (DP2–3) and the caeca longer (DP3+) which has implications for selection of NSPases on the basis of the range or oligosaccharides that they produce.^51^ Thus, feeding an NSPase not only reduces the turmoil during the evolution of the caecal microbiota but also provides energy for the host in the form of SCFA and as a result performance is improved and incidence of enteric disorders reduced.

Recent evidence however suggests that the quantities of AXOS generated by xylanase administration are too small to account for the incremental SCFAs noted in the caecum.^51^, ^57^, ^58^ Thus, the concept of a prebiotic in the true sense of the word is challenged. An alternative hypothesis has been forwarded whereby the enzyme generates small amounts of AXOS which then act as a signal to stimulate production of xylanases and other fibre degrading enzymes by those residents of the microbiota that have such a capacity.^59^, ^60^, ^61^ These species subsequently degrade the fibre structures that are present in the digesta and would otherwise have escaped digestion, producing the additional SCFA observed. Recent evidence has shown that birds that have been grown on a diet containing a xylanase harbour a caecal microbiota which is far better adapted to digest xylan than their counterparts who have never been exposed to a xylanase.^62^ Thus, it seems that NSPases are simply selecting for and stimulating a fibre degrading microbiota by provision of signalling molecules. These bacteria can produce very large quantities of fibre degrading enzymes, as much as an order of magnitude more than is added commercially to the diet. Such a process takes time however and this may explain the significant lag often noted between addition of an enzyme and the response noted. In some situations, the benefit of an NSPase is not seen until the latter half of the production cycle, which coincides with when the caecum is most active. In this regard NSPases and indeed the oligo‐saccharides they generate when added in such small quantities, are not acting as a prebiotic but as what we are now describing as a ‘stimbiotics’. We define a stimbiotic as a compound, often an oligosaccharide, which has the property when added in very small quantities, to stimulate bacteria in both the ileum and caecum to produce their own fibre degrading enzymes and ultimately enhance SCFA production. A stimbiotic can be the same molecule as a prebiotic, it is just the fact that it is fed at concentrations well below those capable of supporting significant fermentation directly. Furthermore, whereas a prebiotic would be expected to be fermented immediately and result in increased SCFA concentrations within hours of feeding, a stimbiotic would not be expected to deliver a similar response for several weeks. Regardless, both a prebiotic and stimbiotic are expected to be used by similar species, e.g. *Ruminococcaceae* and *Lachnospiraceae* in the caeca where butyrate would be the most important product. It is the balance between beneficial products of fermentation such as butyrate and other SCFA, and destructive products such as ammonia, amines, indoles, etc. which determine the health status and hence integrity of the intestinal absorptive surface and the underlying integument.^63^, ^64^ Indeed systemic health, particularly that of the liver, can be especially sensitive to the rate of flow of such metabolites from the intestines^23^ and as such the consequences of poor intestinal health can spill over into challenges at a systemic level as well. Thus, maintenance of good intestinal health has benefits beyond efficient digestion and nutrient absorption and should therefore be a primary goal if general health of the bird is to be optimized.

In the absence of an NSPase there may still be some breakdown of NSP in the ileum by the resident microbiota. As noted earlier, the microbiota of the ileum appear to adapt over a sufficient amount of time and produce xylanases at a level, that to begin with, is only sufficient to dissolve insoluble xylan material but not depolymerize them and as a consequence viscosity increases.^42^, ^59^ Presumably a small proportion of this material is reduced to oligosaccharides and then to monosaccharides to provide energy for the species producing these enzymes. As the bird ages these species become more active and xylanase activity increases resulting in significant further depolymerization of these dissolved, previously viscous arabinoxylans and viscosity falls.^42^ The ileal microbiota is thus responsible for generating a water‐soluble substrate which, if not fermented in the small intestine, flows into the caeca where it will be used as substrate. As the caeca sieve out any particulate matter and even large, soluble carbohydrates, the greater the dissolution of insoluble fibre, and the greater the reduction in molecular weight of the soluble fibre, the greater the potential for fermentation in the caeca. This process of generation of soluble, fermentable xylan from insoluble xylan is markedly accelerated in the presence of an NSPase.^65^ The degree of acceleration depends upon the initial microbiota and its ability to adapt in the absence of the NSPase and hence variation in such a response is to be expected. Furthermore, variability in response to NSPases could well be explained in large part to variation in the amount and size of prebiotic oligosachharides released by different NSPases.^66^

### Phytases

Phytic acid is not only a poorly available form of phosphorus but also a significant antinutrient as it binds minerals and reduces the digestibility of protein and starch.^67^ Many of these minerals are involved in activation of pancreatic proteases and mucosal alkaline phosphatases (zinc), stabilization of mucin (calcium) and reduction of inflammatory responses (zinc).^68^, ^69^ The application of phytases has been shown to dose dependently increase digestibility of many of these minerals and thus play a role in improving intestinal integrity and digestive efficiency. Phytate also interferes with the digestion of energy and most if not all amino acids, particularly those associated with endogenous losses, with these benefits appearing to be greater in young *versus* older broilers^67^, ^70^ simply because the baseline digestibility of nutrients is lower in younger animals. As a result, a similar ‘nutrient removal’ effect on the ileal and caecal microbiota could be assumed to be just as relevant for phytases as it is for NSPases. Indeed, the use of phytase at doses which significantly increased growth rate and efficiency has also been shown to modulate the ileal and caecal microbiotas.^71^, ^72^ However, in the case of the ileal microbiota, the largest effect on diversity and species numbers seemed to relate to the effect of the phytase on phosphorus supply more so than other nutrients. Indeed, the authors suggested that phosphorus supply may play a role in limiting ileal fermentation and this can be overcome either by adding an inorganic phosphorus source or supplementing with a phytase.^71^ Analysis of the caecal microbiota following administration of either a phytase or very high dosages of commercial proteases did result in significant improvements in growth rate and efficiency and pre‐caecal amino acid digestibility, but the changes noted in the structure of the microbiota were quite different, despite their similar effects in limiting protein supply to the caeca. Thus, even in the caeca the effects of phytase on the microbiota may not only relate to its effect in restricting nitrogen flow and perhaps may again be more linked to its effects on phosphorus and calcium supply. Indeed, it has been suggested that in pigs, the use of a phytase increases phosphorus and calcium availability in the ileum to the detriment of the supply of these minerals in the large intestine, and as a result bacterial activity and populations are restricted.^73^ Phytic acid may therefore be a modulator of the health of the intestines more through its effects on mineral supply than on that of macro‐nutrients *per se*.

When phytate is completely dephosphorylated by phytase the end product is inositol monophosphate (IP1), with the last remaining phosphorus being located on the 2 position of the inositol ring.^74^ Feeding very high levels of phytase has repeatedly been shown to increase inositol levels in digesta and blood of both pigs and chickens^75^, ^76^, ^77^, ^78^, ^79^ which suggests that the IP1 end product of phytase action must be dephosphorylated in the small intestine by another phosphatase. It has subsequently been show that intestinal alkaline phosphatase (iALP) taken from jejunal mucosal scrapings is capable of rapidly dephosphorylating IP1 to inositol and that this enzyme appears to be induced by feeding high levels of phytase.^80^ The iALP plays many roles in addition to dephosphorylation of IP1, one such role is the dephosphorylation of bacterial lipopolysaccharide (LPS) and another is the prevention of transepithelial passage by bacteria.^81^ Dephosphorylation of pro‐inflammatory moieties such as LPS and other phosphorylated pathogen associated molecular patterns (PAMPs) by iALP has been suggested as one of the means by which this enzyme preserves intestinal integrity when disease causing organisms are present.^82^ Thus, dephytinization of the diet by use of ‘superdoses’ of phytase can have a beneficial effect on intestinal health by stimulation of the innate immune system through removal of an antinutrient and provision of a substrate for iALP.

### Starch

Starch represents the largest single energy yielding component of almost all poultry feeds. As such the goal is to ensure that it is digested as fully as possible so that energy recovery from the diet is optimized. Starch that is not digested by the end of the terminal ileum is termed resistant starch. There are several forms of resistant starch,^83^ several of which are available for fermentation by micro‐organisms in the lower intestinal tract,^84^ a process which in general results in the generation of acetate and lactate. Lactate can be converted to butyrate in the caeca by some lactate‐utilizing bacteria and can be partly directly absorbed in the small intestine if it is produced there. There is an optimum rate of lactate production however, and if this is exceeded in the caecum then the structure and integrity of the intestine can be compromised due to the low pH generated and the effect excess lactate has on survival of fibre degrading and fermenting species. Indeed, lactic acid accumulation resulting from absorption disorders in the small intestine can lead to caecal acidosis and washout of sensitive fibre‐degrading bacteria.^85^ Indeed the presence of significant quantities of resistant starch has been shown to significantly reduce the capacity of the microbiota to digest other fibre components.^86^ Thus, enzymes which prevent excess starch reaching the terminal ileum can be of significant benefit. Whilst exogenous amylase may come to mind as the most likely candidate to deliver this benefit and some trials have shown benefits,^87^, ^88^ the value of the amylase in the presence of an NSPase and a phytase is not clearly shown yet, partly due to inadequate experimental design.^89^ In most cases exogenous amylase is successful in young or challenged animals where endogenous amylase production is compromised, or in diets rich in ingredients which contain poorly digested starch structures such as beans or potatoes. Nevertheless, some work suggests that even when significant quantities of starch reach the terminal ileum it does not necessarily result in fermentation in the caeca of the chicken,^90^ possibly as a result of the caecal sieves limiting entry of larger particles and thus restricting their entry.

### Protein

As noted earlier, an incomplete digestion of protein provides a ready nitrogen source for the intestinal microbiota, and if this is not balanced with fermentable carbohydrate then its putrefaction will ensue and as a consquence intestinal damage and loss of function.^64^, ^91^ A recent study showed that if pepsin activity in the proventriculus‐gizzard is compromised, it not only results in impaired protein digestibility in the ileum but significantly increased protein fermentation and production of putrefaction products in caeca.^85^ This demonstrated that when endogenous proteases fail to function properly the consequences are a build‐up of toxic protein fermentation products in the distal intestine.

As noted earlier both phytases and NSPases can improve protein digestibility via different mechanisms, and the third class of enzyme with a role to play in this regard is protease. Digestibility studies have shown that in some circumstances protease addition can improve ileal amino acid digestibility^92^ although these results are equivocal.^93^ If proteases are effective then this would be expected to influence the nitrogen flow into the caeca. Some papers have noted changes in the microbiota structure as a result of feeding proteases but this was at levels ten‐fold higher than the commercial dose.^72^ One point to consider is that with NSPases, phytases and proteases all influencing amino acid digestibility, the effect of one in the absence of the others may well be quite different compared with use of all in combination.^93^ Indeed, several publications suggest that whilst the incremental value of inclusion of a second enzyme class may be justified by the scale of the response, it is often the case that the third enzyme addition has little additional beneficial effect.^94^ Nevertheless, the target should be to maximize uptake of amino acids by terminal ileum and, consequently, minimize their bypass to caecum. If/when protein enters the caecum their conversion to toxic metabolites by bacteria can be minimized by ensuring constant concomitant flow of preferred fermentable carbohydrates (e.g. oligosaccharides). As a general rule, bacteria tend to utilize carbohydrates first, and only when they are depleted will they start fermenting protein. In conclusion, exogenous proteases prevent putrefaction if they are able to reduce protein flow to caecum while carbohydrases inhibit putrefaction by providing preferred carbohydrate substrates for caecal bacteria and improving protein digestion further by reducing viscosity. This is an example of two totally different enzyme types having similar overall effects on bacterial functions. The one removing a harmful fermentation substrate and the other providing beneficial substrates, with the end point being improved intestinal health.

## FURTHER POINTS OF SPECIFIC RELEVANCE FOR FEED ENZYMES AND THEIR ROLE IN INTESTINAL HEALTH

### Necrotic enteritis (NE)

Intestinal integrity is very much compromised when the bird suffers from necrotic enteritis (NE) and feed enzymes seem to fall into two distinct categories with regards to their effects on the outcome of this disease due to their effects on calcium supply and intestinal viscosity.

Excess calcium has been shown to markedly exacerbate the severity of NE and this is accentuated when phytase is used in the diet concurrently.^95^, ^96^ The hypothesis is that the excess calcium activates the NetB toxin from *Clostridium perfringens* and potentiates its activity,^95^ resulting in greater enterocyte damage and leaching of contents into the intestinal lumen which provides the amino acids (in particular) which are required for the growth of the causative organism.^97^ Under such circumstances (i.e. high dietary calcium and an NE challenge) the presence of excess phytase can increase the severity of the disease.^95^ Given this result was obtained under severe and artificial challenge conditions, it is not clear whether such an outcome would be less likely to occur naturally in the presence of high doses of phytase since it limits the likelihood of the pathogen taking hold in the first place via its ability to restrict protein flow to the caeca.

The incidence of NE is clearly higher in birds fed viscous grain‐based diets compared with corn‐based diets^98^, ^99^ and use of an NSPase to reduce viscosity even in a corn/soy‐based diet was associated with reduced NE lesion scores.^100^ The theory is that viscosity delays protein digestion and at the same time creates an anaerobic environment in the small intestine which is more conducive to the establishment of significant populations of *Clostridium perfringens*.

### Lysozyme

Lysozyme (also known as muramidase) has been investigated for many years as an antibacterial agent for cleaning surfaces, as an antibacterial wipe and recently in poultry feed as an alternative to antimicrobial agents in the literature^101^, ^102^, ^103^ although results in unchallenged poultry show no benefits as may be expected. The suggested mode of action is lysis of peptidoglycan in the cell walls of Gram‐positive bacteria and as such it would be expected to influence the structure of the microbiota and hence the health of the intestine. However, it has been shown that heat denaturation or chemical reduction of lysozyme does not remove its antibacterial properties *in vitro*, and often enhances them, even though the enzymatic activity is reduced or even removed.^101^, ^102^, ^104^ One hypothesis is that the denaturation of lysozyme unravels the protein and increases its hydrophobicity markedly, and it is this hydrophobic nature of the protein which facilitates penetration of microbial membranes. Commercial sales of this enzyme (as a muramidase) have recently commenced and it does point to an additional function of enzymes *per se*, i.e. direct antimicrobial activity. Such a role, if successful *in vivo*, should augment and synergize with the current feed enzymes which function via very different mechanisms. The challenge is that the effects of lysozyme (and all other ‘gut health’ additives for that matter) should be tested in the presence of the most commonly used incumbent additives, since in the commercial world it is the additive, not the singular, effect of an enzyme which is of interest to the end‐user.

### Mannanase

Galactomannans are considered both viscous and inflammatory, and hence disrupt the integrity of the intestinal tract.^105^, ^106^, ^107^ It is suggested that the mannans mimic PAMPs for poultry and thus their depolymerization by use of mannanases is thought to reduce inflammatory losses.^105^ Indeed some studies have shown a reduction in immune organ weights and serum immunoglobulin A (IgA), IgG and IgM with use of a β‐mannanase and thus reinforced the PAMP hypothesis.^107^ However, the same studies have shown that the response to mannanases is greatest in the older bird^106^, ^108^ which suggests that the benefit may be driven by a prebiotic or stimbiotic route as much as by the removal of a direct irritant/inflammatory agent. No studies have managed to separate the effects of depolymerization of the inflammatory agent *versus* provision of the oligosaccharides which means proof of the inflammatory effect is missing. Moreover, the galactomannan concentration of test diets is often increased through use of ingredients such as guar gum and palm kernel meal which do not represent the mannan structures found in soybean meal, the more commercially relevant substrate for this enzyme.^106^ As such the responses noted to a mannanase in such circumstances may not be replicated in pure corn/soy diets.

Supplementation of the diet with mannan‐oligosaccharides (MOS) is often used as a means to show the prebiotic and bacterial binding effects of the hydrolysis products of mannanases. Great care must be taken in evaluating such research, however, as the source of the MOS is often yeast cell wall material which not only is a different structure^109^ compared with soybean meal‐derived MOS (a‐(1‐6 mannose backbone with a(1‐2) and a(1‐3) linked mannose branches versus B‐(1‐4) mannose backbone with a(1‐6) galactose branches) but yeast derived MOS not only are impure yeast preparations but also insoluble and hence polymeric rather than oligomeric. Thus, yeast cell wall MOS data cannot be used to support β‐mannanase effects in the bird. Consequently, it is still unclear whether the anti‐inflammatory effects noted when β‐mannanase are fed is due to their effects on the structure of the microbiota, and the subsequent effect that this has on the immune system, or whether the effect is direct as suggested earlier.

## CONCLUSIONS

There are many additives which influence intestinal health including probiotics, organic acids, essential oils, yeast cell wall extracts to name a few. Each has its own mechanism of action and even though these may well be independent of one another, it does not mean that their benefits will simply be additive if they are combined. Most studies on the aforementioned additives are done in isolation and thus empirical data quantifying the interactions between these products is extremely limiting. Thus, the end user needs to consider combinations carefully, but the precedent with respect to combinations of enzymes with differing activities and thus modes of action has suggested that such combinations are sub‐additive in effect as the potential for improvement will clearly diminish as more and more products are included. Thus, the value of any product claiming a benefit with regards to intestinal health will very much be influenced by how many other products are included in the test diet and how this compares with commercial reality.

It is not just other additives that need consideration. The growth rate and efficiency responses observed when feed enzymes are employed vary considerably between studies and the scale and direction of response depends upon multiple factors including those relating to dietary ingredients, genetics, husbandry conditions and age.^38^, ^43^, ^110^ These same factors are just as relevant with regards to the responses noted in intestinal health and help explain the variation noted between studies in the literature.

Regardless, the preservation of intestinal health throughout the life of the chicken is a continuous and evolving task. Following the initial challenges post‐hatch which accrue due to poor ‘seeding’ of the intestinal tract, the bird is exposed to a diet which is initially difficult to digest and presents the microbial invaders with a wide range of possible nutrients. Rapid evolution of the digestive tract and its increasing efficiency with age initiates a stress on the microbiota which may or may not prove detrimental to the host. The key goal is to limit the amount of undigested protein which is made available to the microbiota, particularly that resident in the caeca, whilst at the same time optimizing supply of fermentable fibre such that the fermentation patterns are of benefit to the integrity and function of the intestinal tract. Feed enzymes can play a critical role in this regard in that they can limit the flow of undigested nutrients whilst at the same time, generate fermentable fibre from insoluble fibre. Whether this fermentable fibre is functioning as a direct prebiotic, or as a ‘stimbiotic’, as defined in this article, is not important. The key target is to stimulate digestion of fibre which would otherwise be voided, thus extracting energy but also balancing the microbiota to develop into a structure more focussed on fermentation of fibre rather than protein. The benefit of such an evolution of the microbiota over time is clear with regards to animal performance and intestinal integrity and functionality.
